# Engagement with emotional concerns in general practice: a thematic analysis of GP consultations

**DOI:** 10.3399/BJGPO.2023.0202

**Published:** 2024-03-20

**Authors:** Louis Nerurkar, Iris van der Scheer, Fiona Stevenson

**Affiliations:** 1 University College London, Research Department for Primary Care Research and Population Health, London, UK

**Keywords:** general practice, qualitative research, emotional concerns, mental health

## Abstract

**Background:**

Emotional concerns (defined as any expression of low mood, anxiety, or psychosocial stress) are an important part of the biopsychosocial care model used in modern medical practice. Previous work has demonstrated variable engagement with emotional concerns and that improved communication has been associated with reductions in emotional distress.

**Aim:**

To examine how emotional concerns are engaged with during routine GP consultations.

**Design & setting:**

Secondary study using the Harnessing Resources from the Internet (HaRI) database. The available dataset contains 231 recordings from 10 GPs across eight urban and suburban practices recorded in 2017 and 2018.

**Method:**

The dataset was reviewed to identify any consultations containing emotional concerns (as defined as any expression of low mood, anxiety, or psychosocial stress) before being imported into NVivo (version 12) to facilitate thematic analysis and coding. Reflexive inductive thematic analysis resulted in two major themes.

**Results:**

The two main themes were as follows: engagement with emotional concerns as dynamic throughout consultations; and GPs engage with emotional concerns both diagnostically and therapeutically. In theme 1, this dynamism relates to competing areas of focus, immediate versus delayed engagement and reiteration of concerns throughout consultations. Emotional concerns can be engaged with in a similar way to physical concerns (theme 2) using a diagnostic and treatment-based approach; however, in addition to this, therapeutic listening and conversation is utilised.

**Conclusion:**

Awareness of the dynamic nature of emotional concerns within consultations and encouraging engagement with concerns in a flexible and patient-oriented manner may help improve doctor–patient communication. In addition, investigating how GPs and patients build shared understanding around emotional concerns may identify methods to reduce patients’ emotional distress.

## How this fits in

Emotional concerns are common within general practice consultations and engaging with them has been associated with improved patient outcomes. Previous work has focused on barriers and facilitators to engagement with emotional concerns and this study builds on that using a case-study approach. Through review of whole consultations, we highlight the dynamic nature of engagement within a consultation. Moving forward awareness of this dynamic engagement, and the ways in which emotional concerns are engaged with, may improve communication and patient care.

## Introduction

Emotional concerns can be considered as expressions of low mood, anxiety, or psychosocial stress that may be indicative of mental health problems, social issues, or difficult life circumstances.^1^ Previous studies have shown wide variability in how patients raise, and GPs enquire about, emotional concerns and engage with them.^2^

The biopsychosocial care model is now commonly considered a key approach in medical practice.^3^ The model, originally proposed by Engel,^4^ presents health as a combination of biological, psychological, and social factors. Emotional concerns relate to psychological and social factors and interact with physical symptoms, such as chronic pain,^5,6^ headaches,^7,8^ and have a complex relationship with medically unexplained symptoms.^9–11^ There is evidence of the importance of engaging with emotional concerns to improve patient satisfaction^12^ and patient adherence to treatment.^13^ Other work has also demonstrated that improving doctor’s communication is associated with reduced Global Health Questionnaire scores up to 6 months later,^14^ and exploring and understanding emotional concerns can be used to build a therapeutic alliance and relationship.^15–17^

Engagement with emotional concerns is important. First, emotional concerns may be clues to underlying psychological and physical issues, and through careful engagement can lead to changes in patient beliefs about their illness.^11^ Second, engagement with emotional concerns has been shown to aid the building of a doctor–patient relationship and facilitates treatment acceptance and adherence.^18^ Finally, through emotional engagement, there can be a therapeutic healing relationship that can, in and of itself, be beneficial to the patient.^19^ Therefore, engaging with emotional concerns is important for both diagnosis and treatment of new problems and managing chronic conditions. Further, recent work by Beach *et al* has highlighted how explicit acknowledgement of emotional concerns is associated with reduced consultation length.^20^ Therefore, understanding how emotional concerns are engaged with is not just important for providing high-quality care but also for time management in a highly pressured primary care environment.

Previous studies into emotional concerns have often focused on specific aspects of the consultation, such as the outcomes of consultations, or analysis of GP communication techniques, or diagnostic accuracy.^1^ In contrast, this study examined how emotional concerns were engaged with throughout whole consultations, building on previous work to understand the contextual nature of engagement rather than just the point at which emotional concerns are raised. Further, we also examined how patients raised and engaged with emotional concerns.

## Method

### HaRI database

The Harnessing Resources from the Internet (HaRI) database is a collection of 281 consultations (of which 231 are available for reuse) recorded in 10 practices, which vary according to social deprivation and the sex and ethnic group of the GP in London and the South East of England in 2017 and 2018 (further details of the main study can be found in Seguin *et al*).^21^ The study was able to examine emotional concerns being raised in consultations with a psychological focus as well as within routine GP appointments. The sex and ethnic group of patients for the 50 included studies can be seen in Table 1.

**Table 1.** Sex and ethnic group data for the patients in the 50 included studies

Demographic	*n*	(%)
**Sex**		
Male	35	(70%)
Female	15	(30%)
**Ethnic group**		
White English	40	(80%)
White Irish	1	(2%)
White Other	3	(6%)
Black Caribbean	1	(2%)
Asian Pakistani	1	(2%)
Asian Other	1	(2%)
Mixed Other	1	(2%)
Other (not stated)	1	(2%)
Not stated	1	(2%)

Transcripts in the HaRI database are classified according to International Classification of Primary Care (ICPC) codes. All 231 transcripts were reviewed to identify any consultations in which emotional concerns were raised by either patient or doctor. Emotional concerns were defined as any expression of low mood, anxiety, or psychosocial stress. This set was then compared with the list of consultations coded as containing a psychological or social problem. Any consultations coded as containing a psychological or social problem that had not been identified in the initial review for emotional concerns were reviewed again to ensure content relating to emotional concern was not missed. In total, 50 consultations (21.6%) were identified for inclusion in the thematic analysis.

### Coding and thematic analysis

Transcripts were imported into NVivo (version 12) software for analysis. LN initially used qualitative content analysis to generate key codes and themes across the 50 consultations, which were identified as containing content relating to emotional concerns. After initial coding, codes and constructed themes were discussed in the research team.^22^ The research team consisted of a GP trainee with an interest in communication and mental health (LN), a medical sociologist with expertise in communication and qualitative research (FS), and a doctoral researcher with an interest in health communication (IvS).

The process of group discussion and reflection was used three times to construct the themes presented. Parts of the consultation with discussion of physical symptoms without emotional concerns were coded as 'physical focus' with no further detail. Sections where emotional concerns were present were subject to more detailed coding examining the expression of concerns and responses. Codes used included 'mind–body language' and 'social contributors'. A key code that recurred was 'therapeutic listening and conversation', coded when the doctor allowed the patient time to talk about an emotional concern without interruption or redirection, or with verbal cues demonstrating engagement with the concern.

Team discussions concluded that data were best presented as case studies to demonstrate the incremental nature of the construction and communication about mental health concerns between GPs and patients.

## Results

Data were organised according to the following two main themes: (i) engagement with emotional concerns as dynamic throughout consultations; and (ii) GPs engage with emotional concerns both diagnostically and therapeutically (Box 1). The themes are illustrated using two case studies. The first theme demonstrates the dynamic nature of the consultations (theme 1) and is illustrated through extended examination of the interactions between patient and doctor within a single consultation. The way that concerns are engaged with (theme 2) links to this dynamic engagement as the forms of engagement change throughout and between consultations. These two case studies were chosen as they provide examples of all the points illustrated in Box 1 and demonstrate differing approaches to engagement found in the 50 consultations reviewed. The sum of immediate and delayed engagement with emotional concerns is less than the total number of 50 cases as in some cases there was no engagement.

Box 1 **Major themes and sub-themes of thematic analysis of GP consultations. (*n* = X ) refers to the number of cases this was observed in**

**1. Engagement with emotional concerns as dynamic throughout consultations**

1.1. GP engagement with emotional concerns can be immediate (*n* = 28) or delayed (*n* = 7)

1.2. Patient’s emotional concerns may be reiterated throughout a consultation

1.3. Physical and practical concerns may take precedence over emotional concerns

**2. GPs engage with emotional concerns both diagnostically and therapeutically**

2.1. By assessment of diagnostic criteria, social and lifestyle contributors, and risk management

2.2. Through therapeutic listening and conversation

2.3. Through treatment discussion, offers, and reassurance

To highlight how the codes and themes raised relate to the cases below, we have annotated the text with the numbers from Box 1 to allow for cross-referencing to the codes and themes without disrupting the flow of the case studies; for example, where a patient reiterates emotional concerns would be annotated as [1.2]. Extracts are labelled with their case study number (1 or 2) and extract number; for example, 1.1, 1.2, and so on.

### Case study 1

In this case study a patient presenting for the first time to the GP raises several ongoing issues (Table 2, extract 1.1). There is a mixture of psychological symptoms, including emotional concerns, and physical symptoms presented early in the consultation.

**Table 2.** Extract 1.1

Line
Pt: I’m really sorry but I would like to see you lots and lots in the near future because I feel I’ve got lots of things going on that I’ve probably put to the back of my mind and I just would like to see somebody regular. Erm, I went to the doctors about four, five, six weeks ago and said basically, “I don’t cope ever so well in life. And I’m quite anxious. And, erm, I’m shouting a lot at my children and it’s affecting life.” Erm, I had CBT a few years ago, about eight years ago as I said. **Dr: Mmm.** Pt: I get headaches all the time. It- That’s one of the catalysts, so I really want to get my headaches sorted out and I would like to be put forward for some CBT. And the doctor that I saw gave me some drugs, which I said, “I really don’t want to be on anti-depressant drugs.” *[… further conversation about psychological referral and ongoing symptoms…]* And then I’m irritable and then I’m— And I just don’t know what to do. **Dr: And then it’s this circle.** Pt: And hormonal headaches is my big thing. **Dr: I think what probably— because—** Pt: (Sighs) **Dr: Obviously because we’ve never met before, I suspect the best thing today is that you** **just tell me a bit about yourself.** Pt: Okay. **Dr: So that I can be clear because we’re going to have to make a list of things that we’re** **going to cover.**

The patient begins by stating she has a series of concerns that she would like addressed by a single GP over more than one consultation (lines 1–3). The inclusion of emotional concerns is made clear *'I’ve got a lot of things going on*…' (line 2), *'I don’t cope ever so well in life. And I’m quite anxious*' (lines 4–5). CBT (cognitive behavioural therapy) is mentioned twice (lines 6 and 9) alongside the physical symptom of headaches (line 9), which is redone as '*hormonal headaches*' (line 14). After allowing the patient space and engaging in therapeutic listening as the patient summarises their problems [2.2], the GP states an agenda for the consultation of finding out more about the patient and prioritising their concerns (lines 17–21).

Following this extract, the doctor summarises the issues raised, and the patient raises her dad’s death 3 years ago (not shown here), highlighting a possible social contributor. Subsequently, the doctor restates the need to understand more about the patient’s life (lines 17–18), exploring social contributors (Table 3, extract 1.2).

**Table 3.** Extract 1.2

Line
**Dr: Yeah. I under— I understand. So, if you just tell me. I think it will be helpful to tell me a** **little bit about life now.** Pt: Yes. **Dr: So I’m clear what’s going on now.** Pt: Okay, okay. *[… 53 second segment where patient talks about home life…]* Nothing is wrong and I really mean that. I’ve got a really kind of good deal **Dr: So, you have a very happy life and a lovely supportive family and friendships.** Pt: Yeah, yeah. So, I don’t know what’s wrong with me. I’ve got lovely friends. It’s my big, big birthday in a few weeks and loads of people are making a fuss and it’s— You know. I’m lucky. I consider myself lucky and I guess part of my anxiety is I wonder when it’s going to break. **Dr: Yeah, yeah. And I think that’s very normal anxiety for people whose life seems to almost** **sometimes have a charmed life.**

This section highlights how social contributors [2.1] may be explored when emotional concerns are engaged with, this extract can be considered as a therapeutic conversation [2.2] as the GP only enquires specifically about things to demonstrate active listening (lines 5–11). There is also evidence of shared understanding being built, when the GP summarises about a happy life and supportive relationships and the patient agrees (lines 8–9). At the end the GP provides a reassurance statement [2.3] to the patient about her emotional concern (anxiety) (lines 12–13). This process of therapeutic listening was one of the most common techniques observed across several GPs and consultations.

The doctor then asks a series of questions examining the patient’s psychological symptoms in more detail asking about sleep, the issues that cause anxiety, and possible symptoms of obsessive compulsive disorder (OCD) before moving on to if the patient still enjoys things. The consultation then reaches a point where physical symptoms are raised and directly linked to emotional concerns first by the patient then the doctor.

This extract (Table 4, extract 1.3) highlights how dynamic shifts in focus can be a result of both patient and doctor. First, the patient raises the abdominal pain when she discusses work, the doctor then asks *'are those the days you go to work?*' exploring a possible psychological link (lines 4–7). Then through acknowledging the previously raised issue of headaches the doctor shifts the focus of the consultation on to this symptom (line 13).

**Table 4.** Extract 1.3

Line
**Dr: What kinds of things do you enjoy?** Pt: My friends, my family. **Dr: Okay** Pt: And I do enjoy work when I get there. But I always on a Wednesday and a Friday morning I have— So, I have a gluten intolerance and I always have a tummy ache on Wednesday and Friday morning. **Dr: Are those the days you go to work?** Pt: Yeah. So, I— But once I’ve gone, I’m all right, but actually, I’d rather stay at home and have a cup of tea with my friends or something. You know, wouldn’t we all. But, erm, so I know that anxiety triggers me. And so, over the years I’ve had a test for coeliac disease. I’m not coeliac. I'm definitely intolerant to gluten. I can handle a bit. If I eat too much or I’m anxious, I’m in trouble. But I can handle that. I’m all right with that. That’s not one of my problems now. **Dr: Okay. Now, the last thing I think we’ll have time to talk about this morning is just your headaches.**

In this consultation there is a shifting focus as the doctor explores issues the patient has raised, initially the patient is given space to raise issues before the doctor engages with some of the emotional concerns presented by discussing the patient’s social situation and the symptoms of her anxiety. The patient highlights abdominal pain when talking about work, which is presented as a physical problem, but also is linked, by both the doctor and patient, to anxiety. This is followed by pursuit by the doctor of the physical symptom of headaches.

Overall, this consultation illustrates extensive engagement with the patient’s emotional concerns. However, there is still a balance with the physical (headaches) and at times emotional concerns are not engaged with extensively (abdominal pain and anxiety). This highlights that even when emotional concerns are the primary focus of the consultation, there is still a dynamic balance with physical symptoms. Crucially, both doctor and patient influence this balance.

### Case study 2

In this second case study the patient presents talking about something going on '*in my head*' referring to an ear, nose, and throat (ENT) symptom that is under investigation. In the first extract from this consultation the patient talks about a recent ENT appointment and the possibility of an magnetic resonance imaging (MRI) scan.

In this extract (Table 5, extract 2.1) the patient begins by using language that could indicate emotional concern, such as '*the only thing that’s driving me mad*' and '*it is getting me down really*', when discussing a problem going on '*in my head'* for which they have seen an ENT consultant (lines 2–3). Later in the consultation, as the patient talks more about their anxiety (see below), it becomes clearer that these statements could refer to physical or emotional concerns. However, at this point there is a focus on the practical issue of referral for MRI (lines 11–12) [1.3].

**Table 5.** Extract 2.1

Line
**Dr: How are you doing?** Pt: Yeah just, the only thing that’s driving me mad is all this in my in my head like we said you know before. So um I think you got a letter from [name of clinic] **Dr: You went to see [consultant’s name], didn’t you?** Pt: That’s that’s right and um he said it could possibly be an inner ear benign tumour **Dr: Right** Pt: So I need an MRI scan um I’ve got an appointment for ear, nose and throat on the 28th of this month. But then obviously, there would still be a period of time, wouldn’t there, to, but it is, it is getting me down really. I don’t know if you can refer me any quicker or it’s not something you do **Dr: We generally don’t refer people for MRI scans um ‘cause ah, there are a few** **circumstances we can** Pt: Yeah *[… discussion about ENT referring for MRI and ENT appointment…]*

Following discussion about practical aspects of upcoming appointments,(Table 6, extract 2.2) there is a series of exchanges in which emotional concerns are presented by the patient, with some reference to physical issues, with responses by the doctor focused on the physical concerns.

**Table 6.** Extract 2.2

Line
Pt: Yeah so that’s all all going through. But I’ll I’ll be honest with you, it is worrying the life out of me you know it’s sort of frightening, isn’t it? But um yeah so that’s those really um and I didn’t **Dr: That’s for the aortic valve, isn’t it?** Pt: Yeah that’s right, yeah, yeah. I’m not going out at all I haven’t, since I’ve come back from hospital, I had that appointment about a month and I said to the doctor about exercise.. he said, “Yeah, just just be careful,” but in my mind, I was starting to get out for a walk, which was clearing my head a little bit really **Dr: Mmm** Pt: Um but I mean is it safe just to gently walk or **Dr: What would be your worry, what would you wor’ what?** Pt: Just if I’m damaging or hurt *[Dr shakes head]* No? **Dr: No** Pt: No, that’s brilliant *[…]* Pt: You know. Um but I do find that I’m not going out, I’m getting asked out by my sisters to go for something to eat and I’m not going, because, even though I try to see the grandchildren, it’s just pulling me down, you know just, it’s hard to explain just the, you know having to be somewhere for a couple of hours with all this in your head. And even today, which I’ve never missed an appointment with you, never, or any appointments. But I really felt like just ringing and saying I can’t make it. I felt terrible. Um and also, I need a hearing aid apparently **Dr: Yeah, I’ll be honest with you, I haven’t referred you for the hearing aid** Pt: Yeah I don’t, I don’t think it is because it gets.. when I get anxious you know like I’ve got to go out to the hospital or I’ve got to come down here, it gets worse **Dr: Does it?** Pt: My hearing gets worse and this feeling in my head gets worse **Dr: So you’re not that keen to have a hearing aid or?** Pt: I would rather find out exactly what’s causing it *[…]* Pt: And I wanted to talk to you, how do you feel, if it is anxiety, because it definitely is, I can feel myself, you know, getting real stressed having to go out the door. And it’s getting worse. I mean is it something, I mean I don’t take any tablets apart from my you know blood pressure and the *[doctor smiles]* but I mean I don’t, I don’t *[patient laughs]* **Dr: Apart from these** Pt: Yeah, yeah. Yeah I mean I’m on that new cresta um **Dr: Yes** Pt: Yeah **Dr: Getting on alright on that?** Pt: I’m still aching everywhere. Yeah, but I didn’t notice any

These segments together highlight how engagement with emotional concerns can be reiterated throughout a consultation [1.2]: *'it’s just pulling me down*' (line 18), '*all this in your head*' (line 19), and *'I can feel myself, you know, getting real stressed'* (line 30–31). Despite this reiteration the GP focuses more on the practical, discussing the hearing aid referral (line 27) and the tablets (line 34) [1.3], this contrasts with a discussion about walking with an aortic valve problem where the GP does provide reassurance with regard to a physical problem (lines 11–14).

After a brief segment about recent blood tests, the GP acknowledges some of the previously raised emotional concerns and signposts the patient towards support.

In this section (Table 7, extract 2.3) the GP acknowledges that the patient has previously raised emotional concerns [1.1], specifically anxiety, throughout the consultation (line 1). They signpost the patient to a self-help leaflet and contacts for counselling (lines 10–11), as the patient asks '*without tablets or'* (line 12) and then shows hesitation '*yeah um*' (line 14) the GP enquires directly if the patient wants tablets (line 15) [2.3]. The patient responds in the negative but seeks reassurance that it’s '*common*' to feel as they do given their circumstances (line 16) and receives reassurance (line 17). The GP does not ask any further questions about the symptoms themselves and focuses on management of the symptom including providing reassurance at the end (lines 17–19) [2.3].

**Table 7.** Extract 2.3

Line
**Dr: So coming back to what you were saying about the anxiety** Pt: Yeah, yeah **Dr: and feeling that that may be a part of what you’re experiencing** Pt: Yeah because I’ve been stuck in **Dr: Yeah** Pt: You know, if it wasn’t for this in my head, I’d be laughing, I think, all the time, you know, and hoping about and **Dr: So, there are there are things we can do.** *[… Doctor provides patient with self-help leaflets and signposting about anxiety…]* **Dr: If you want to think about sort of counselling type you can also contact them, and you** **can either do it by phone or online** Pt: Without tablets or **Dr: Yeah** Pt: Yeah um **Dr: Well now did you do you want tablets?** Pt: No, no, no not if um would it quite common because I’ve been stuck in all these months? **Dr: Absolutely** Pt: Yeah **Dr: Yeah drives people mad**

Overall, in this example the patient raises emotional concerns numerous times alongside physical concerns but these are not engaged with by the GP at the time. Instead, the GP takes a more physically and practically focused approach. Towards the end of the consultation the GP shifts the focus to the emotional concerns providing signposting and reassurance. Thus, although emotional concerns may be raised at different points in a consultation, even when not explicitly acknowledged, they may still be ‘heard’ and returned to.

These segments contrast with the first case study where the GP asked further questions and engaged with emotional concerns the patient had, encouraging them to elaborate. Together these case studies demonstrate how emotional concerns can be raised, moved away from, and then reoccur in a dynamic manner throughout consultations. Throughout the data engagement varied from therapeutic listening to acknowledgement and refocusing of the consultation, as well as absence of acknowledgement.

## Discussion

### Summary

In these two case studies we demonstrate how emotional concerns can be engaged with throughout GP consultations and how this engagement can vary both within and between consultations. Emotional concerns are often engaged with immediately, but engagement may be delayed particularly in the presence of physical and/or practical concerns. Emotional concerns were engaged with in a variety of ways, including therapeutic conversation, reassurance, and discussion of treatment, both pharmaceutical and not. The depth of engagement with emotional concerns, particularly through therapeutic conversation, was often influenced by other competing physical and practical concerns.

### Strengths and limitations

This study is strengthened by using consultations that have both physical and psychological presenting complaints. The data are snapshots, and comments on historical or future consultations cannot be made. There is no access to the GP notes, prescribing information, or follow-up information, so no comment can be made on the effect of these discussions on outcomes or further engagement with interventions. Inclusion of interview data exploring ideas about these discussions would have allowed a different perspective to the evaluation of consultations.

Differences in the expression of emotional concerns has been shown among ethnic groups in both the US^23^ and The Netherlands,^24^ given that this study had predominantly participants from a White English ethnic group, its results should be considered within that context.

### Comparison with existing literature

Across the data we observed variable engagement with emotional concerns, both within individual consultations and between consultations, mirroring the findings of previous work in this area, which has reported variable use of medical and psychosocial questions, as well as differences in responses centred on emotions.^2^

Work looking at emotional concerns has often focused on facilitators and barriers to engagement with emotional concerns and competing issues that influence depth of engagement.^1^ However, the challenge in these consultations is more complex than just increasing engagement. Determining what is an appropriate level of engagement is difficult and this is highlighted in the above examples of the dynamic discussion that occurs within consultations. Physical symptoms taking precedence,^1,25^ time limitations,^1^ and confidence^26^ may influence the level of engagement in a consultation. For example, headaches can be a symptom of underlying pathology and impact quality of life, therefore warranting further discussion. On the other hand, abdominal pain linked to anxiety and gluten — *'that’s not one of my problems now*' — may not, highlighting how engagement is not only determined by the doctor but also the patient. In both the case studies presented there are expressions of emotional concerns early in the consultation. Work by Gafaranga and Britten found that the development of a consultation is one of dynamic construction by both patient and GP.^27^ Early expression of emotional concerns in these consultations suggested a negotiation occurring as to possible agenda items that the GP can engage with.

Theme 2, which is GPs engage with emotional concerns both diagnostically and therapeutically, shows how emotional concerns are often used to identify or explore problems further (diagnostics) by both patient (linking anxiety to worsening ear problems in case study 2) and doctor (exploring social situations and the relation to anxiety in case study 1). We also observed different approaches to therapeutic engagement. In case study 1 there is extensive therapeutic conversation (extract 2 and 3) in a process where shared understanding is built. In case study 2 there is signposting about resources for anxiety, which is a therapeutic outcome although different to case study 1. Finally, in both case studies reassurance was used to manage emotional concerns. Poulson *et al* also identified normalisation of concerns, a form of reassurance, to be a method used by GPs in consultations.^25^ Parker *et al* have discussed a similar model to theme two and argue the doctor can be seen as detective (diagnostic), drug (therapeutic relationship), and collaborator (shared understanding).^17^

### Implications for research and practice

Through awareness of how emotional concerns can be raised and engaged with dynamically, GPs can consider their influence and utilise them as diagnostic clues and therapeutic tools. Previous work has demonstrated how therapeutic connection and empathy are tools employed by GPs in addressing emotional concerns.^28^ Moving forward prospective interviews and/or focus groups with GPs linked to consultation data would help examine how GPs view the management of emotional concerns.

The key points for clinicians to consider in their practice include the following: (i) if emotional concerns are not engaged with they may recur throughout a consultation; (ii) physical and practical concerns can take precedence, but returning to emotional concerns may be beneficial; (iii) if given space to discuss concerns, patients themselves may emphasise those of most importance to them.

## Data

Some of the transcripts are available for reuse, please contact the original PI of the study Fiona Stevenson (f.stevenson@ucl.ac.uk) for more details.
